# Seeking shelter from the storm: Conservation and management of imperiled species in a changing climate

**DOI:** 10.1002/ece3.5277

**Published:** 2019-05-30

**Authors:** Susan C. Walls, William J. Barichivich, Jonathan Chandler, Ashley M. Meade, Marysa Milinichik, Katherine M. O'Donnell, Megan E. Owens, Terry Peacock, Joseph Reinman, Rebecca C. Watling, Olivia E. Wetsch

**Affiliations:** ^1^ Wetland and Aquatic Research Center U.S. Geological Survey Gainesville Florida; ^2^ St. Marks National Wildlife Refuge U.S. Fish and Wildlife Service St. Marks Florida; ^3^ Environmental Stewards Program Conservation Legacy Durango Colorado

**Keywords:** adaptation strategies, *Ambystoma cingulatum*, climate change, coastal wetlands, frosted flatwoods salamander, Hurricane Michael, imperiled species management, saltwater inundation, specific conductance, storm surge

## Abstract

Climate change is anticipated to exacerbate the extinction risk of species whose persistence is already compromised by habitat loss, invasive species, disease, or other stressors. In coastal areas of the southeastern United States (USA), many imperiled vertebrates are vulnerable to hurricanes, which climate models predict to become more severe in the 21st century. Despite this escalating threat, explicit adaptation strategies that address hurricane threats, in particular, and climate change more generally, are largely underrepresented in recovery planning and implementation. We provide a basis for stronger emphasis on strategic planning for imperiled species facing the increasing threat of catastrophic hurricanes. Our reasoning comes from observations of short‐term environmental and biological impacts of Hurricane Michael, which impacted the Gulf Coast of the southeastern USA in October 2018. During this storm, St. Marks National Wildlife Refuge, located along the northern Gulf of Mexico's coast in the panhandle region of Florida, received storm surge that was 3.0–3.6 m (NAVD88) above sea level. Storm surge pushed sea water into some ephemeral freshwater ponds used for breeding by the federally threatened frosted flatwoods salamander (*Ambystoma cingulatum*). After the storm, specific conductance across all ponds measured varied from 80 to 23,100 µS/cm, compared to 75 to 445 µS/cm in spring 2018. For 17 overwashed wetlands that were measured in both spring and fall 2018, posthurricane conductance observations were, on average, more than 90 times higher than in the previous spring, setting the stage for varying population responses across this coastal landscape. Importantly, we found live individual flatwoods salamanders at both overwashed and non‐overwashed sites, although we cannot yet assess the demographic consequences of this storm. We outline actions that could be incorporated into climate adaptation strategies and recovery planning for imperiled species, like *A. cingulatum*, that are associated with freshwater coastal wetlands in hurricane‐prone regions.

## 1 INTRODUCTION

A species' viability is shaped by local and landscape scale processes that reflect three fundamental conservation principles, collectively known as “the 3Rs” (Shaffer & Stein, 2000; Smith et al., 2018; U.S. Fish & Wildlife Service [USFWS], 2016). At the local scale, *resiliency* refers to the ability of individual populations to withstand environmental and demographic stochasticity. At the landscape scale, a viable species has sufficient *representation* across a variety of ecological settings, enabling it to adapt to changing environmental conditions over space and time. Lastly, a species' viability depends on *redundancy*, the existence of multiple populations at both scales, minimizing extinction risk due to catastrophic events. Most species of conservation concern are deficient in one or more of these three measures, but those that have few, small, isolated populations with limited geographic ranges are especially at risk (Purvis, Gittleman, Cowlishaw, & Mace, 2000; Soulé, 1987), as “no single population is immune to the chance of catastrophic extinction” (Shaffer & Stein, 2000).

Climate change is already negatively impacting many taxa—including global fisheries that are critical food resources for a growing human population (Free et al., 2019; Pacifici et al., 2017). Consequently, climate change is anticipated to be one of the most significant drivers of ecological and societal change in the coming century (Lawler et al., 2009). For freshwater coastal wetlands, accelerated sea level rise and saltwater intrusion have been the focal points of climate change research and management planning (Osland et al., 2016). However, tropical cyclonic storms (hurricanes) also pose a formidable threat to coastal ecosystems. Since the 1970s, both frequency and intensity of the strongest North Atlantic tropical cyclones have increased (Bhatia et al., 2019; Hartmann et al., 2013). Current climate change models (e.g., the CMIP3–A1B scenario and the CMIP5‐early and late 21st century scenarios) predict fewer Atlantic tropical storms in warmer climates and an increase in the frequency of the most intense hurricanes (Categories 4 and 5), although this predicted increased frequency varies among models (i.e., a substantially increased frequency is predicted for Categories 4 and 5 hurricanes using the CMIP3 model, but smaller increases are projected using the CMIP5‐early and CMIP5‐late models; Knutson et al., 2013). This projected escalation of catastrophic hurricanes is an unprecedented challenge for conservation and management of imperiled species that inhabit coastal ecosystems.

**Figure 1:**
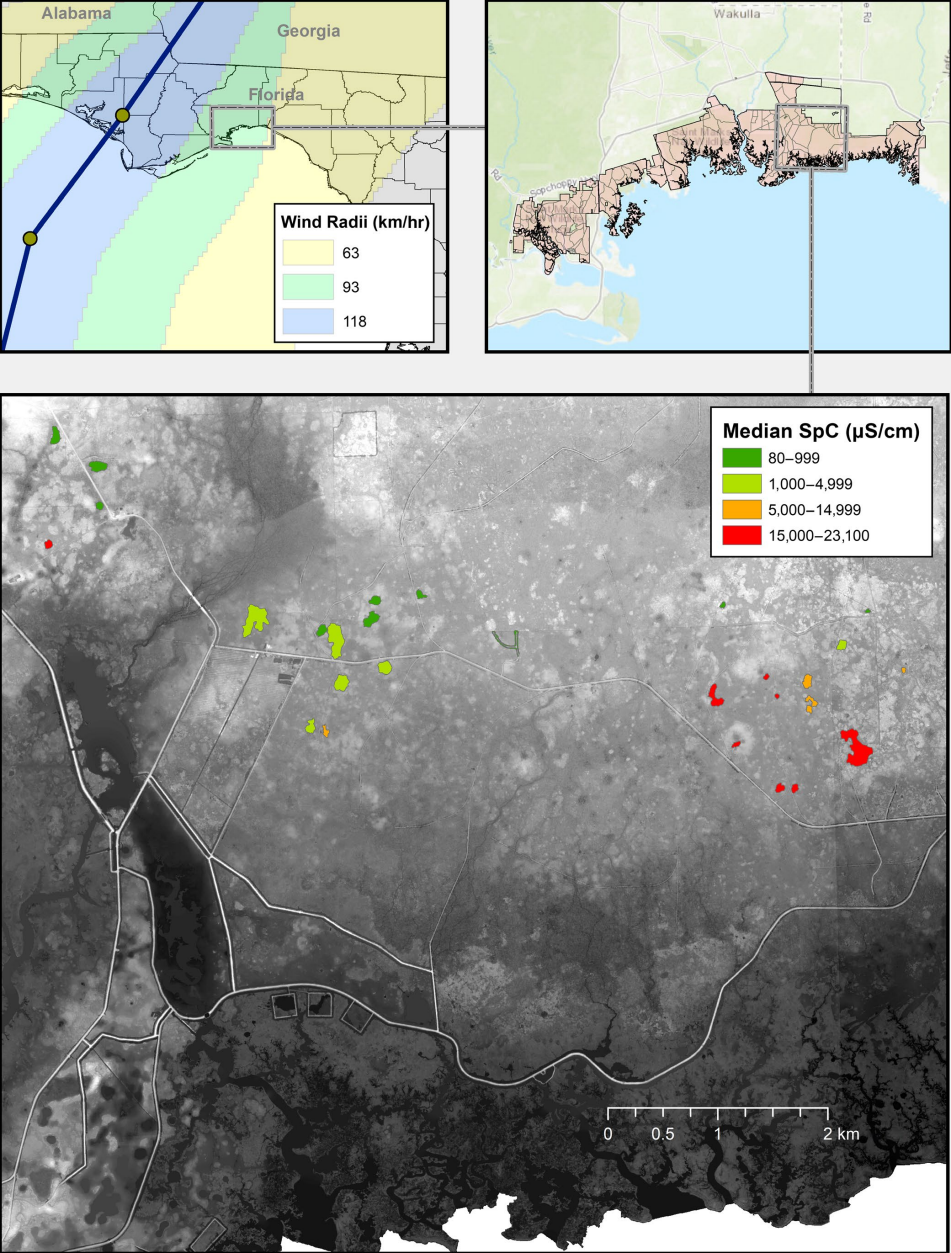
Upper left: Location of St. Marks National Wildlife Refuge (box) in the panhandle region of Florida, USA. Solid dark blue line indicates the path of Hurricane Michael and its associated windfields (from NOAA National Hurricane Center, https://www.nhc.noaa.gov/gis/). Upper right: Region of the Refuge (box) in which frosted flatwoods salamanders are found (the eastern‐most St. Marks Unit). Bottom: *Ambystoma cingulatum* breeding wetlands (*n* = 29) from which we measured water chemistry. The extent to which storm surge and its associated saltwater intrusion affected these wetlands is depicted by wetlands (polygons) of different colors

Freshwater wetlands provide critical habitat for a diverse array of organisms, including many sensitive species of conservation concern (Howard et al., 2015). For example, flatwoods salamanders (*Ambystoma cingulatum* and *Ambystoma bishopi*) are specialists of mesic pine flatwoods and savannas on the Coastal Plain of the southeastern USA; these two species currently exist only as isolated metapopulations in a few locations within their historic range (Semlitsch, Walls, Barichivich, & O'Donnell, 2017). St. Marks National Wildlife Refuge (hereafter, “the Refuge”), located on the Gulf Coast of Florida, USA (Figure 1), has been a stronghold for the federally threatened frosted flatwoods salamander (*A. cingulatum*), with at least 42 ponds actively used for breeding in at least one year between 2013 and 2018 (USFWS, 2019a). Because of their proximity to the coast, these wetlands are extremely vulnerable to storm surge and its associated saltwater flooding.

The impacts of hurricane‐related saltwater intrusion have been well documented for wetland vegetation and many vertebrates found in coastal habitats (e.g., Guntenspergen et al., 1995; Gunzburger, Hughes, Barichivich, & Staiger, 2010; Roman et al., 1994; Schriever, Ramspott, Crother, & Fontenot, 2009; Steyer et al., 2010), yet proactive approaches to reduce threats from hurricanes *before* they occur are often lacking. This deficiency provides an opportunity to revisit existing strategies for managing vulnerable species in hurricane‐prone areas and to develop or update action plans where needed (sensu Povilitis & Suckling, 2010).

We provide preliminary assessments of some environmental and biological impacts from storm surge associated with Hurricane Michael. On 10 October 2018, this storm made landfall in the panhandle region of Florida and resulted in saltwater inundation in some areas of the Refuge, including many wetlands used by *A. cingulatum* for breeding. We also explore the extent to which hurricane‐adaptation strategies have been developed for imperiled vertebrates in hurricane‐prone coastal areas. Based on our assessments, we outline the need for a proactive management framework to reduce risk of future catastrophic storm impacts on vulnerable populations of this species, in particular, and for imperiled species in hurricane‐prone regions, in general.

## 2 St. MARKS NATIONAL WILDLIFE REFUGE, FROSTED FLATWOODS SALAMANDERS, AND HURRICANE MICHAEL

St. Marks National Wildlife Refuge is a wetland‐rich region in one of the five richest biodiversity hotspots in North America (Blaustein, 2008; Noss et al., 2015). The Gulf Coast of the southeastern USA is also extremely vulnerable to sea level rise (Enwright, Griffith, & Osland, 2016). The Refuge consists of 32,042 ha of upland and wetland habitats broadly classified as sandhills, flatwoods, and hammocks. A diverse amphibian community consisting of 31 species (20 anurans, 11 salamanders) can be found on the Refuge (Dodd, Barichivich, Johnson, & Staiger, 2007). *Ambystoma cingulatum* is confined to hydric flatwoods located in the eastern‐most region of the Refuge, the St. Marks Unit (Figure 1).

**Figure 2:**
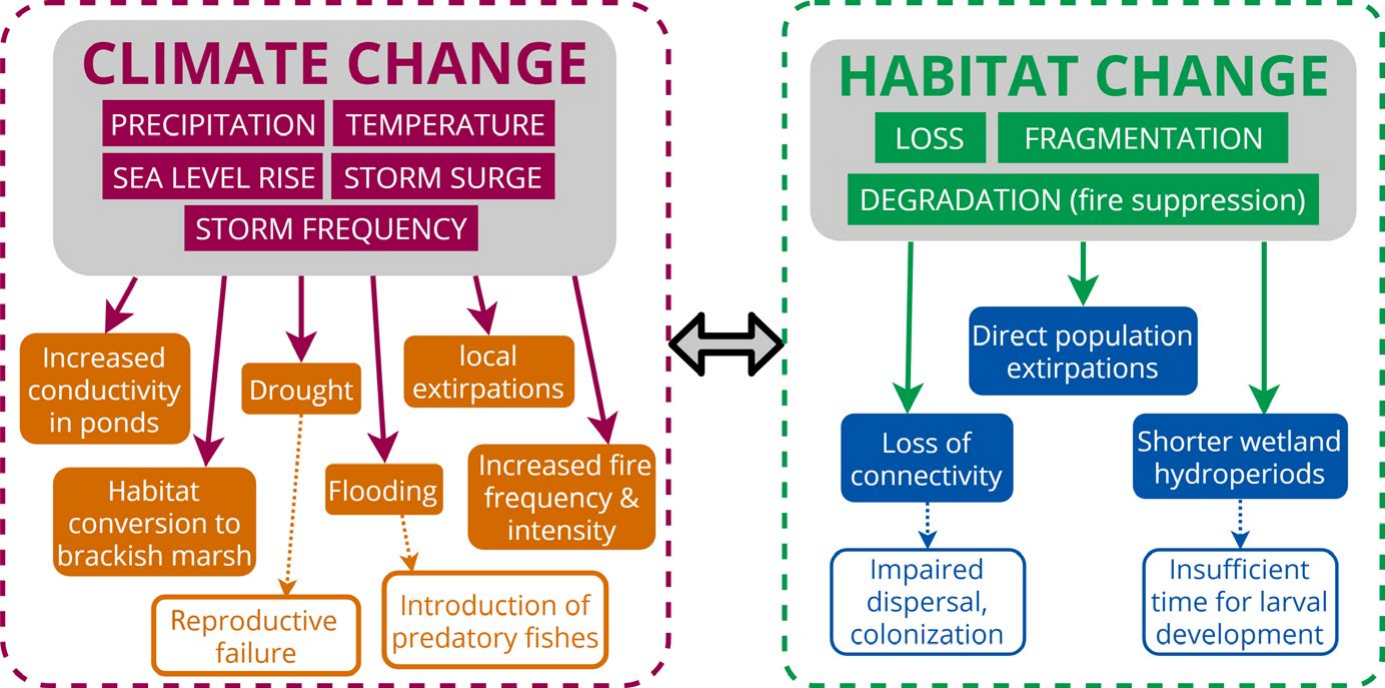
Two of the principal stressors, changes in climate and habitat, that impact populations of the frosted flatwoods salamander, along with their ecological and demographic consequences. Dashed lines represent examples of indirect effects from key consequences. The double arrow between the two sets of consequences indicates that changes in climate and habitat interact synergistically to compound the negative effects of each stressor individually

**Figure 3:**
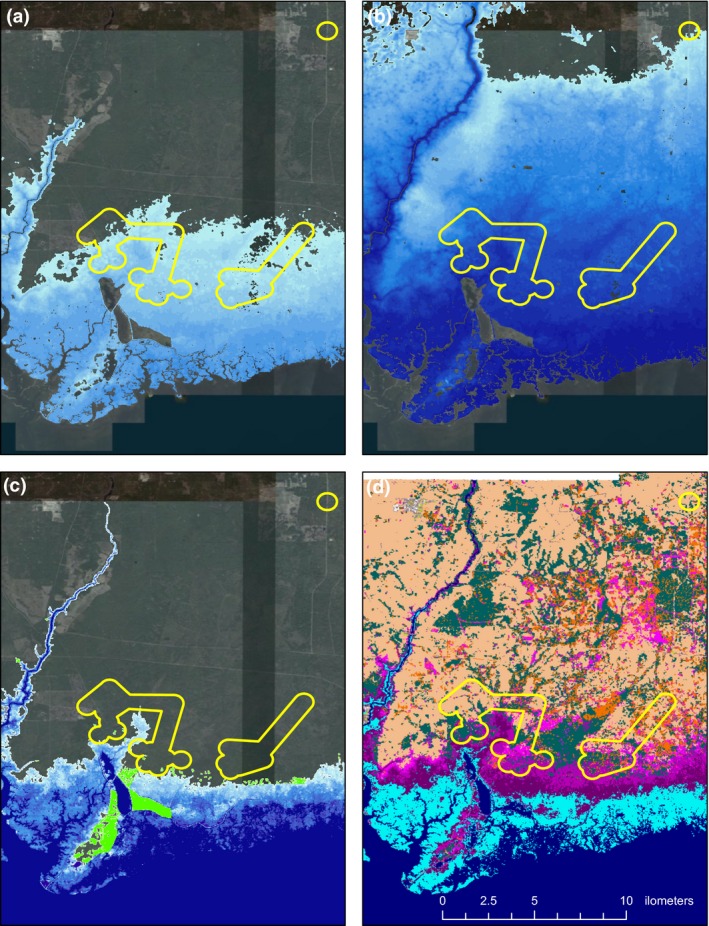
SLOSH models of maximum surge level during a (a) Category 1 and (b) Category 3 storm. (c) Areas of inundation (in light blue) under a scenario of 1 m of sea level rise for Critical Habitat Units (yellow polygons) FFS‐3A (bottom left polygon), and FFS‐3B (bottom right polygon) at SMNWR. (Critical Habitat Unit FFS‐3C [upper right] does not occur within the Refuge boundaries). (d) Areas converted to marsh habitat (in pink) as a consequence of 1 m of sea level rise

Historically, flatwoods salamanders occurred across southern Alabama, the panhandle and north central regions of Florida, southern Georgia, and South Carolina (Palis & Means, 2005). In recent years, however, and despite intense efforts to monitor and locate new populations, the two flatwoods salamander species have collectively experienced an 86.8% loss of populations (Semlitsch et al., 2017). The primary threats currently affecting flatwoods salamanders are changes in habitat (loss, fragmentation, and degradation), climate (particularly drought and variation in the timing of rainfall), and interactions between these threats (Figure 2). Extreme precipitation events, resulting in drought and flooding, along with hurricane‐induced storm surge (Lin, Lane, Emanuel, Sullivan, & Donnelly, 2014), can potentially impact amphibians that use freshwater coastal wetlands (Walls, Barichivich, & Brown, 2013). Moreover, phenological shifts in the timing of key climatic events (e.g., pond‐filling and drying) can have significant consequences for individual survival and species persistence (Walls et al., 2013, and references therein). Lastly, sea level rise threatens coastal freshwater wetlands, their surrounding upland habitats, and landscape connectivity (Benscoter et al., 2013; Leonard et al., 2017; Tebaldi, Strauss, & Zervas, 2012; Wahl, Calafat, & Luther, 2014; Woodruff, Irish, & Camargo, 2013). Because of proximity to the Gulf of Mexico and potential exposure to hurricane‐related storm surge and sea level rise, the St. Marks Unit of the Refuge (approximately 2.4 m above sea level) harbors the most susceptible *A. cingulatum* Critical Habitat Units (Units FFS‐3A and FFS‐3B; Figure 3). The use of sea level rise and marsh mitigation data from National Oceanic and Atmospheric Administration (NOAA, 2018a) in a SLOSH (Sea, Lake, and Overland Surges from Hurricanes) model (NOAA, 2018b) predicts that, in a Category 1 storm, maximum storm surge could inundate all but a couple of breeding sites in FFS‐3A and FFS‐3B (Figure 3a). In a Category 3 storm, maximum storm surge could completely inundate all breeding sites in FFS‐3A and FFS‐3B and could approach the southern boundary of Critical Habitat Unit FFS‐3C (located north of the Refuge; Figure 3b). In more intense storms (Categories 4 and 5), the maximum surge level could completely inundate FFS‐3C as well (not shown).

Figure 3c shows the predicted outcome of 1 m of future sea level rise in this area. Under this scenario, sea levels would approach Critical Habitat Units FFS‐3A and FFS‐3B and would inundate a few breeding sites. However, perhaps the most dramatic occurrence would be the advancement of marsh habitat in front of encroaching sea levels (Figure 3d): the habitat that encompasses most of the current freshwater wetlands in these units would change to brackish marsh, conditions that would presumably be incompatible with persistence of flatwoods salamanders.

Hurricane Michael made landfall at Category 5 intensity on the Saffir–Simpson scale, with a maximum sustained wind speed of 160 mph (257 kph) and a minimum pressure of 919 mb (Beven, Berg, & Hagen, 2019). This storm rapidly intensified in the 24 hr preceding landfall and was one of only four Category 5 hurricanes to make landfall in the United States, with projected peak storm surge inundation values as high as 4.3 m above ground level (AGL) along the Gulf of Mexico coast (Beven et al., 2019). A U.S. Geological Survey (USGS) survey team measured a high water mark of 2.4 m AGL (3.6 NAVD88; USGS, 2018) within the Refuge (Beven et al., 2019), and a high water mark close to our study ponds indicated storm surge reached 3 m (NAVD88) above sea level in that area (USGS, 2018). This storm surge was exacerbated by counter clockwise winds that pushed water from the Gulf of Mexico as much as 6.4 km inland, swamping at least 17 freshwater ponds used by *A. cingulatum* with salt water (Figure 1, Table S1; USFWS, 2018a). These ponds were the only ones we were able to measure; assuming other ponds located between these 17 were also inundated, the total number of inundated ponds would be at least 35. Although this water would normally recede within approximately a day, the area in which many of these ponds are located was covered with seawater for at least a week because of an elevated tram road south of the ponds (Figure 1), which impeded the outward flow of water.

**Figure 4:**
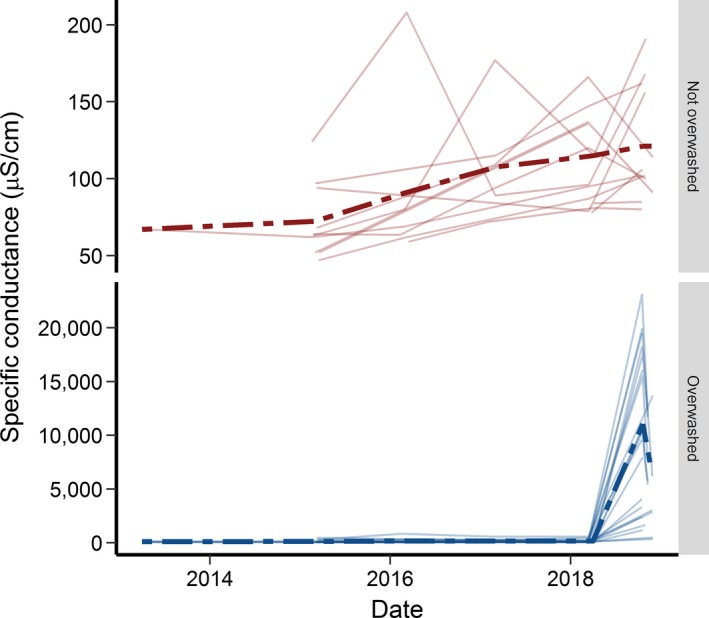
Specific conductance (µS/cm) of non‐overwashed ponds and overwashed ponds at St. Marks National Wildlife Refuge. Each line represents an individual site over time. Dotted lines are mean values of specific conductance

From 16 October to 30 November 2018, we measured specific conductance (SpC) using a HydroLab Quanta water quality meter during each visit to each wetland according to standard USGS protocols (USGS, 1997 to present). We report the median of five water quality readings at each site (Table S1). We identified overwashed wetlands by comparing poststorm SpC levels to prestorm levels (before the storm, a single measurement of SpC was taken per site each spring). As defined by Gunzburger et al. (2010), overwashed wetlands had higher specific conductance than non‐overwashed wetlands by several orders of magnitude (Figure 4). In March and April 2018, SpC (µS/cm) of 27 breeding ponds known to be used by *A. cingulatum* ranged from 75 to 445 µS/cm across all ponds. Soon after the storm (16–29 October), SpC of these same ponds ranged from 80 to 23,100 µS/cm (Figure 4; Table S1). For the 17 ponds that were overwashed, conductivity increased by  a factor of 11.2–216.7 (mean ± 1 *SD* = 92.8 ± 65.2) per pond, whereas it only changed by a factor of 0.8–2.0 (mean ± 1 *SD *= 1.2 ± 0.4) for the remaining 10 ponds that were not overwashed (Table S1). For ponds sampled more long‐term (from April 2013 to March/April 2018), mean per‐season SpC (for overwashed and non‐overwashed ponds combined) ranged from a low of 88 µS/cm to a high of 150 µS/cm. But, in October 2018, the mean median value increased to  7,556 µS/cm, a 50‐ to 86‐fold overall increase for all ponds combined. A map of poststorm SpC values reveals the spatial extent of storm surge for ponds inhabited by *A. cingulatum* (Figure 1). This map provides a refinement of the actual spatial extent of saltwater intrusion as well as a basis to compare predictions from SLOSH models for this area (Figure 3 a,b).

After the hurricane, we opportunistically observed (during systematic trapping and assessments of storm damage) both terrestrial and wetland‐associated species and noted their status (alive or dead). We confined our observation period of dead individuals to 15 ‐ 19 October 2018 to avoid potentially double counting individuals, to infer that the cause of death was possibly storm‐related, and because after this time dead animals were scavenged or otherwise significantly less identifiable. Except for *A. cingulatum*, for which we report captures through 10 December, we recorded observations of live individuals through 26 October, with most individual amphibians observed during a nocturnal search on 25 October. We observed numerous unidentified individual fishes (both alive and dead), live crayfish, and 15 species of both amphibians and reptiles (live and dead combined; Table 1). We searched for *A. cingulatum* at two breeding sites that did not get inundated by storm surge and continued to monitor three sites that are part of a long‐term capture‐mark‐recapture study. Prior to the storm, these latter sites were equipped with partial drift fences and funnel traps designed to intercept salamanders migrating to and from breeding ponds. For all three sites combined, a marked population of 888 individuals (including 442 head‐started metamorphs) existed as of May 2018. All three existing study sites were inundated by storm surge during Hurricane Michael, and the storm destroyed or severely damaged most of the drift fences, compromising our ability to detect migrating salamanders. Nevertheless, we erected partial, temporary fences and set out traps (wire‐mesh funnel traps) along them at all five sites (two new sites and three existing ones), checking them daily to determine whether salamanders were still present at these sites.

**Table 1:** Species opportunistically observed at both overwashed and non‐overwashed sites post‐Hurricane Michael at St. Marks National Wildlife Refuge, FL, USA

Common name	Species	Count	
		Alive	Dead
Invertebrates			
Crayfish	Unidentified	3	
Fishes	Unidentified	Numerous	24
Amphibians			
Frosted Flatwoods Salamander	*Ambystoma cingulatum*	36^a^	
Two‐toed Amphiuma	*Amphiuma means*		7
Dwarf Salamander	*Eurycea quadridigitata*	4^b^	
Eastern Newt	*Notophthalmus viridescens*		4
Cricket Frog	*Acris gryllus*	Numerous	
Oak Toad	*Anaxyrus quercicus*	2+	
Southern Toad	*Anaxyrus terrestris*	2	
Eastern Narrow‐mouthed Toad	*Gastrophryne carolinensis*	2+	
Cope's Gray Treefrog	*Hyla chrysoscelis*	1	
Green Treefrog	*Hyla cinerea*	Numerous	
Pinewoods Treefrog	*Hyla femoralis*	3	
American Bullfrog/Pig frog	*Lithobates catesbeianus/L. grylio*		4
Pig Frog	*Lithobates grylio*	3	
Southern Leopard Frog	*Lithobates sphenocephalus*	Numerous	7
Little Grass Frog	*Pseudacris ocularis*	4	
Unidentified frog			3
Unidentified toad			1
Reptiles			
Cottonmouth	*Agkistrodon piscivorous*	3+	1
Red‐bellied Mud Snake	*Farancia abacura*		1
Eastern Diamond‐backed Rattlesnake	*Crotalus adamanteus*	2	
Pygmy Rattlesnake	*Sistrurus miliaris*	10+	
Ribbon Snake	*Thamnophis sauritus*	2	
Rough Green Snake	*Opheodrys aestivus*	1	
Black Racer	*Coluber constrictor*	1	
Unidentified snake			2
Green Anole	*Anolis carolinensis*	1	
Five‐lined Skink	*Plestiodon fasciatus*	1	
Stinkpot	*Sternotherus odoratus*	1	1
Yellow‐bellied Slider	*Trachemys scripta scripta*	2	
Florida Softshell Turtle	*Apalone ferox*	2	
Chicken Turtle	*Deirochelys reticularia*	1	
Mud Turtle	*Kinosternon subrubrum*	1	
American Alligator	*Alligator mississippiensis*	Numerous	1 small
Total		81+	50+

Note

All dead animals were observed in overwashed areas, whereas we observed live animals at both overwashed and non‐overwashed sites. Counts of dead individuals were limited to 15–19 October 2018; those of live individuals were observed through 26 October. Observations of live *A. cingulatum* are from 25 October to 4 December 2018.

a Includes five gravid females and 10 individuals recaptured from a previous year, all from overwashed sites.

b One individual swimming in water at a known overwashed site.

**Figure 5:**
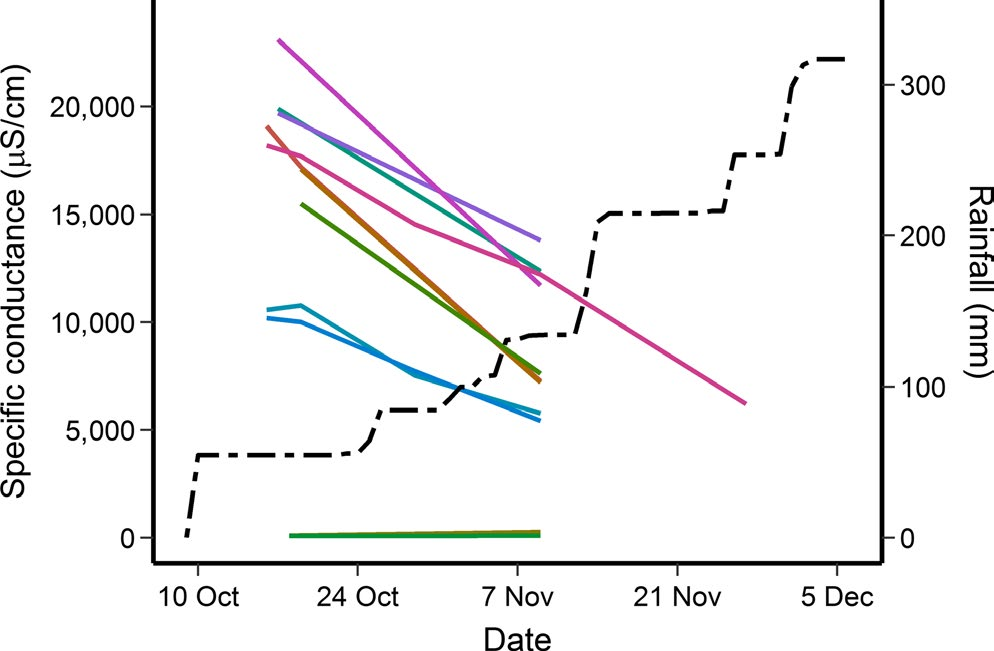
Amount of rainfall (black dotted line) recorded since 10 October 2018 at the St. Marks (East) station of the Western Regional Climate Center's (WRCC) RAWS USA Climate Archive (2018). Colored lines represent specific conductance at each of 11 individual ponds. Horizontal colored lines represent two non‐overwashed ponds

Importantly, as of 10 December 2018, we had captured 36 live adult flatwoods salamanders, all but one of which was captured in traps at drift fences (one was found underneath debris in shallow water). These captures represented 34 unique individuals: 10 individuals were recaptured from previous seasons and two were recaptured within the fall 2018 season. These captures were at both overwashed and non‐overwashed sites and included five gravid females ready to lay eggs. Although at least some adult individuals survived the storm, the potential effects of salt‐contaminated soil and pond water on terrestrially deposited eggs and aquatic larvae are unknown for this species. Therefore, it is not yet possible for us to estimate the demographic consequences of this storm. Continued sampling of 11 ponds after the storm indicated that SpC declined over an 8‐week period, but this decline could reflect dilution of pond water because >300 mm of rainfall fell in the area during that time (Figure 5).

We are continuing to monitor these breeding ponds and, in February and March 2019, we captured only 11 aquatic larvae during 4,940 trap‐nights at three of 60 ponds surveyed. In contrast, in spring 2018, we captured 532 larvae during 3,340 trap‐nights at 23 of 58 ponds surveyed. These results indicate a 71.5‐fold decrease in larval captures per trap‐night effort and a decrease from 40% of ponds occupied (not adjusting for imperfect detection) to only 5% in 2019. Although not conclusive, these results suggest that juvenile recruitment was extremely low in 2019 following the storm, perhaps because developing embryos and aquatic larvae are more sensitive to salinity than are adults. Additionally, in 2018 above‐normal rainfall occurred in summer preceding the onset of fall salamander breeding migrations and continued into early winter, encompassing the peak migration period (e.g., from May to December, this region received 126.4 cm of rainfall in 2018 compared to 80.5 cm in 2017; Western Region Climate Center [WRCC], 2018). The timing and amount of rainfall during these months are critical to reproductive success of flatwoods salamanders: the above‐normal rainfall this region received in summer 2018 did not allow these normally ephemeral ponds to dry, which allowed aquatic predators (e.g., fishes, crayfish, and insects) of larval salamanders to persist. These hypotheses need to be tested empirically to tease apart the interacting effects of salinity, hydroperiod, and predation on different life history stages of this species.

We hypothesize that there will be differences in salamander vital rates across the Refuge: not all wetlands were inundated by storm surge and specific conductance varied among those that were (Figure 1; Table S1). Moreover, although the ability of *A. cingulatum* to tolerate salinity is not known, many species of amphibians are known to occur in saline habitats (Hopkins & Brodie, 2015; Hossack et al., 2018). For one species (the Rough‐skinned Newt, *Taricha granulosa*), individuals occupying a coastal stream on the Pacific Coast of North America varied in their physiological tolerance to elevated salinity, with individuals closer to the ocean exhibiting less of a response to salt stress than those found farther upstream (Hopkins et al., 2016). Physiological plasticity is a significant means by which species may persist across varying environmental conditions (Chevin & Hoffmann, 2017; Herrando‐Pérez et al., 2018; Novarro et al., 2018). Ultimately, plasticity may buffer some species from extinction by promoting physiological, behavioral, and/or other adaptations that could minimize potentially lethal exposure to stressors such as salinity (Chown et al., 2010; Riddell, Odom, Damm, & Sears, 2018; Sergio, Blas, & Hiraldo, 2018; Urban, Richardson, & Freidenfelds, 2014).

## 3 NEED FOR A PROACTIVE CLIMATE CHANGE ADAPTATION PLAN

Species' declines and extinctions are predicted to escalate as changes in land‐use, climate, and other stressors intensify (Selwood, McGeoch, & Mac Nally, 2015). Many conservation plans focus on the impacts from mean, long‐term climatic changes but fail to consider changes in the frequency and intensity of extreme weather and climatic events (Maxwell et al., 2019). Moreover, compared to climate change mitigation and adaptation plans developed for human communities, few management strategies have been developed specifically for imperiled species, and ones targeting amphibians are especially rare (Appendix S1A). Many actions that are designed for human communities, such as construction of flood levees and dikes, could be modified appropriately to protect biodiversity. The few climate adaptation actions that have been explored for amphibians primarily focus on mediating effects of temperature increases and more variable precipitation (Griffis‐Kyle, 2016; Shoo et al., 2011). Muths and Fisher (2017) pointed out a similar lack of action planning in response to the amphibian decline crisis. Consequently, Grant et al. (2017) issued a call for improved responses to the potential threat of emerging infectious diseases, a significant factor in the declines and extinctions of amphibian species.

As Grant et al. (2017) stressed with the threat of emerging infectious diseases, effective conservation and management in the face of climatic uncertainty require a proactive framework to reduce risk of future catastrophic storm impacts to vulnerable populations of imperiled species. In its 4th Assessment Report, the Intergovernmental Panel on Climate Change classified adaptation strategies as those consisting of *protective* actions (those that increase robustness by building species and/or habitat resilience to disturbance events), *accommodating* options (those that increase flexibility), and *retreat* options (those that enhance adaptability) (Nicholls et al., 2007). For amphibians in coastal wetlands, appropriate protective actions may include (a) minimizing or eliminating landscape features that are conduits for future inundation by plugging ditches and installing water control structures; (b) restoring habitat and maintaining its suitability using prescribed fire and other tools; and (c) engineering protective structures such as sea walls, flood levees, dikes, culverts, and impoundments for flood abatement (Enwright et al., 2016; Noble et al., 2014). Examples of accommodating actions include (a) enhancing population resilience (e.g., increasing genetic diversity), (b) increasing connectivity among populations, and (c) acquiring land to allow future managed translocations. Although building resilience is fundamental to future conservation of populations in coastal environments, more active planning and management will likely be needed to protect the most vulnerable coastal environments (Fisk, Haines, & Toki, 2017). Meeting this need may require implementing a range of retreat measures, including assisted migration or managed translocation, establishment of ecological corridors, and ex situ conservation (Fisk et al., 2017; Noble et al., 2014).

Of 381 threatened and endangered species (Endangered Species Act of 1973) that occur in the southeastern USA, half (191 species) may be found in coastal regions that are vulnerable to the impacts of hurricanes (Appendix S1B, Tables S2 and S3). Of 65 species of listed vertebrates, six currently have no formal recovery plan (with a seventh, Bachman's Warbler, exempt from recovery planning). Many others have static, outdated recovery plans (i.e., 8–36 years old)—the status of many recovery plans, in general (Malcom & Li, 2018)—that do not consider recent projections of hurricane threats (Appendix S1B, Table S3). For example, only 33 vertebrate species in this region have recovery plans that mention hurricanes and, of those, the need for management actions in response to the threat of hurricanes is discussed for only five (St. Andrew beach mouse, red‐cockaded woodpecker, guajon, reticulated and frosted flatwoods salamanders) and, to a limited extent, for three others (a total of 12% of all vertebrate species considered). For several species, hurricanes are secondary threats compared to others (e.g., habitat loss, fragmentation, and degradation), which may partially explain why the threat of hurricanes is not addressed in their recovery plans. For others, the potential impacts of hurricanes do not appear to be thoroughly discussed or documented with supporting data. For example, in the Cape Sable Seaside Sparrow (*Ammodramus maritimus mirabilis*) recovery plan, the USFWS (1999) stated that “declines in population numbers were detected following Hurricane Andrew; however, a leveling off of declines, or rebound in population numbers, would be expected if populations were recovering from a *single adverse event* [italics ours], such as Hurricane Andrew. Instead, declines continued as would be expected under continuing adverse hydrological conditions.” In another example, the Recovery Plan for the Loggerhead Sea Turtle (*Caretta caretta*) stated that “sea turtles have evolved a strategy to offset these natural events by laying large numbers of eggs and by distributing their nests both spatially and temporally,” despite reporting losses of up to 54% of entire nests in various tropical storm systems that occurred from 1985 to 2001 (USFWS, 2009).

A need to improve preparation for climate change in recovery planning is not unique to the United States; mention of specific actions to address climate change is relatively uncommon in recovery plans in Australia as well (Hoeppner & Hughes, 2018). In part because of the difficulty in keeping recovery plans up‐to‐date, the U.S. Fish and Wildlife Service (USFWS) has revised its approach to recovery planning and implementation (USFWS, 2017). The agency's framework currently consists of three integrated components—a Species Status Assessment, the Recovery Plan, and a Recovery Implementation Strategy (RIS) for each listed species. Unlike past approaches, the RIS facilitates adaptation to changing circumstances and, thus, more flexibility in recovery strategies (USFWS, 2017). This new, stream‐lined framework is built upon the 3R platform, and its adoption provides an opportunity to integrate new approaches to buffering against catastrophic events like hurricanes. However, given that 30% of 1,660 currently listed species are still in need of a recovery plan, and the USFWS National Listing Workplan prioritizes >550 other species that are awaiting status reviews and listing determinations, it is unclear when existing recovery plans may be reviewed for possible updating (USFWS, 2018b). Even once developed, implementation of such actions is generally slow and remains a significant challenge to conservation and management of imperiled species in a changing climate (Stein et al., 2013).

## 4 CONCLUSIONS

Globally, coastal wetlands are among those anticipated to be the most severely impacted by climate change because of increased frequency and intensity of coastal storms, as well as increased flooding and secondary salinization from sea level rise (Albecker & McCoy, 2017). It is too early to determine the full impact that Hurricane Michael may have had on freshwater wetlands and their associated biodiversity at St. Marks National Wildlife Refuge. Our detection of live individuals of several species and, especially, of *A. cingulatum* following saltwater inundation of breeding habitat is encouraging in the short‐term, but we remain cautious in our expectations for long‐term resiliency of this vital population.

Information on this species' tolerance of salinity, the short‐term effects of the storm on immediate mortality, and longer‐term effects on population viability are lacking. It is premature to assess whether any populations were extirpated because of this storm, which will require several years of poststorm monitoring (Adams et al., 2013; Gibbs, Droege, & Eagle, 1998). It would also be extremely difficult to detect storm‐related mortality in individuals of this small‐bodied amphibian, which do not linger in the environment very long after death or are readily scavenged by predators. Moreover, these fossorial animals probably were not active on the surface at the time of the storm's impact, and therefore, mortality may have occurred in their burrows or under vegetation that we could not search. The options we outline for developing hurricane‐adaptation plans are relevant to flatwoods salamander populations at St. Marks National Wildlife Refuge, as well as to other ecological communities in coastal areas. The alternative option—to do nothing or to maintain the status quo—until we do know more about these topics is a high‐risk strategy for an imperiled species with low resiliency and very little redundancy of populations across its range. In such circumstances, one catastrophic event (natural or otherwise) could eliminate, or at least seriously compromise, all remaining populations of a given species (e.g., the extinction of the Golden Toad, *Bufo periglenes*, which coincided with an exceptionally dry interval caused by an El Niño event: Anchukaitis & Evans, 2010).

For *A. cingulatum*, reducing vulnerability to hurricanes and related storm surge events will ultimately depend on the existence of resilient, redundant, and representative populations throughout this species' historical range. Both species of flatwoods salamanders are experiencing severe population losses, and, of those that remain, resiliency is likely low to moderate (USFWS, 2019a). In terms of redundancy, *A. cingulatum* currently exists only as isolated metapopulations in a few locations within its historical range (Semlitsch et al., 2017). The USFWS considers there to be only two representative units, one of which contains only one known breeding site of moderate resiliency (USFWS, 2019a). Should that population become extirpated and no other natural populations are found in this unit, then losing that population would mean the loss of an entire representation unit (USFWS, 2019a). The remaining representation unit would include St. Marks National Wildlife Refuge and Apalachicola National Forest, both of which are considered relative strongholds for occupancy by *A. cingulatum*. Prior to the Hurricane Michael, the Refuge had many redundant populations of moderate resiliency (USFWS, 2019a). Our continued monitoring of Refuge populations will allow us to assess whether wetlands that were overwashed during the hurricane continue to support frosted flatwoods salamanders. In addition, the Refuge has recently expanded its northern boundary through land acquisition and is partnering with state and other federal government agencies to restore habitat to suitable conditions within this area. Eventually, captive‐reared flatwoods salamanders could be translocated to restored wetlands to the north, as outlined in the species' Draft Recovery Plan (USFWS, 2019b). Ultimately, the development and implementation of a climate and, specifically, hurricane‐adaptation strategy will help ensure the long‐term persistence of this species in the face of a changing climate.

## CONFLICT OF INTEREST

None declared.

## AUTHOR CONTRIBUTIONS

WB, JC, AM, MM, KO, MO, RW, and OW made field observations and collected data. SW wrote the manuscript and handled revisions from all coauthors. All authors commented on the manuscript, contributing critically to the final version of the study. All authors gave final approval of the manuscript for publication.

## Data Availability

Data are deposited in U.S. Geological Survey's ScienceBase (https://doi.org/10.5066/P9V4A2GV).
