# Supplementary material for: Seeking shelter from the storm: Conservation and management of imperiled species in a changing climate
Source: Ecol Evol. 2019 May 30;9(12):7122–33. doi: 10.1002/ece3.5277 (PMC6662284; doi:10.1002/ece3.5277)
Supplement: Supplementary file 1 [file ECE3-9-7122-s001.docx]

**Supporting Information**

Table S1. Specific conductance (SpC, in µS/cm) for 27 flatwoods salamander breeding ponds that were sampled both before Hurricane Michael in March/April 2018 and after (16-29 October 2018). Only one measure per site was taken each date in spring 2018, whereas median values (of 5 subsamples per pond) are reported for the first post-hurricane sample of each pond in October 2018.

| Pond | Date 1 | | | | SpC | Date 2 | | Median SpC | | Factor Change | |
| --- | --- | --- | --- | --- | --- | --- | --- | --- | --- | --- | --- |
| **Overwashed ponds** | |  | |  | | |  | |  | |  |
| 4 | 3/15/2018 | | | | 77 | 10/18/2018 | | 10,560 | | 137.1 | |
| 101 | 3/13/2018 | | | | 93 | 10/29/2018 | | 1,650 | | 17.7 | |
| 107 | 3/21/2018 | | | | 159 | 10/17/2018 | | 3,330 | | 20.9 | |
| 108 | 3/4/2018 | | | | 144 | 10/17/2018 | | 9,610 | | 66.7 | |
| 110 | 4/5/2018 | | | | 113 | 10/17/2018 | | 4,080 | | 36.1 | |
| 129 | 3/15/2018 | | | | 259 | 10/19/2018 | | 16,100 | | 62.2 | |
| 132 | 4/5/2018 | | | | 96 | 10/17/2018 | | 17,300 | | 180.2 | |
| 142 | 3/15/2018 | | | | 144 | 10/17/2018 | | 19,900 | | 138.2 | |
| 145 | 4/5/2018 | | | | 110 | 10/19/2018 | | 10,780 | | 98.0 | |
| 1003 | 3/13/2018 | | | | 99 | 10/19/2018 | | 15,500 | | 156.6 | |
| 1008 | 3/21/2018 | | | | 75 | 10/19/2018 | | 1,180 | | 15.7 | |
| 1601 | 4/5/2018 | | | | 96 | 10/16/2018 | | 10,190 | | 106.1 | |
| 1604 | 4/5/2018 | | | | 81 | 10/19/2018 | | 7,960 | | 98.3 | |
| 2001 | 3/15/2018 | | | | 138 | 10/17/2018 | | 1,550 | | 11.2 | |
| 2002 | 3/15/2018 | | | | 445 | 10/17/2018 | | 19,500 | | 43.8 | |
| 2003 | 3/13/2018 | | | | 135 | 10/17/2018 | | 23,100 | | 171.1 | |
| 3001 | 4/5/2018 | | | | 84 | 10/16/2018 | | 18,200 | | 216.7 | |
| **mean** |  | | | | **138** |  | | **11,205** | | **92.8** | |
|  |  | | | |  |  | |  | |  | |
| **Non-overwashed ponds** | | |  | |  |  | |  | |  | |
| 16 | 3/15/2018 | | | | 79 | 10/29/2018 | | 156 | | 2.0 | |
| 18 | 3/13/2018 | | | | 147 | 10/17/2018 | | 162 | | 1.1 | |
| 103 | 3/13/2018 | | | | 123 | 10/18/2018 | | 99 | | 0.8 | |
| 1001 | 3/15/2018 | | | | 87 | 10/29/2018 | | 102 | | 1.2 | |
| 1002 | 4/5/2018 | | | | 115 | 10/18/2018 | | 106 | | 0.9 | |
| 1014 | 3/15/2018 | | | | 95 | 10/17/2018 | | 102 | | 1.1 | |
| 1041 | 3/27/2018 | | | | 78 | 10/18/2018 | | 106 | | 1.4 | |
| 1605 | 4/5/2018 | | | | 81 | 10/17/2018 | | 80 | | 1.0 | |
| 1701 | 4/5/2018 | | | | 84 | 10/17/2018 | | 85 | | 1.0 | |
| 3014 | 3/15/2018 | | | | 96 | 10/29/2018 | | 168 | | 1.8 | |
| **mean** |  | | | | **98.5** |  | | **116.6** | | **1.2** | |

Appendix S1A.

On 8 November 2018, we conducted a literature search using ISI Web of Knowledge and the search terms “climate change mitigation strategy” and “climate change adaptation strategy.” For each of these, we also refined the search using the terms “biodiversity” and “amphibian”. These searches returned the following number of publications:

| **Search Term** | **General (no refinement)** | **“Biodiversity”** | **“Amphibian”** |
| --- | --- | --- | --- |
| climate change mitigation strategy | 2,841 | 213 | 5 |
| climate change adaptation strategy | 5,012 | 477 | 19 |

Regardless of the search term used, the trends were similar; i.e., the number of publications that mentioned strategies for biodiversity are an order of magnitude less than those that discuss either class of strategy more generally. In both searches, only a handful of publications referenced amphibians, specifically.

Appendix S1B.

**METHODS**

We searched the U.S. Fish & Wildlife Service Environmental Conservation Online System (ECOS) <https://ecos.fws.gov/ecp0/reports/ad-hoc-species-report?status=E&status=T&status=EmE&status=EmT&status=SAE&status=SAT&lead=4&header=Southeast+(Region+4)&fleadreg=on&fstatus=on&finvpop=on>, accessed 7 November 2018) for currently listed species in the southeastern United States (USFWS Region 4). We then used each species range, as indicated in each species profile, to assess whether a species potentially occurred in hurricane-prone regions of the Caribbean, Gulf of Mexico coasts, and the Atlantic coast. We then used the ECOS system to determine whether a species has an existing recovery plan and, if so, the status of that plan (whether it was a final version or a draft and the year in which it was prepared). If a recovery plan existed, we searched each one to determine whether the plan contained a strategy for enabling the species to adapt to the threat of hurricanes and associated impacts such as storm surge.

**RESULTS**

In the southeastern United States, a total of 381 species of plants, invertebrates and vertebrates are currently listed under the Endangered Species Act as either Threatened or Endangered, with another 4 listed because of their similarity of appearance to a threatened taxon. Of these, 191 species (50.1 %) occur in coastal regions that are vulnerable to the impacts of hurricanes (Tables S2 & S3).

Table S2. Federally-listed plant and invertebrate species that occur in hurricane-prone regions of the Caribbean and the southeastern United States. E = Endangered; T = Threatened; S = Similarity of appearance to a threatened taxon.

| **Scientific Name** | **Common Name** | **Federal Listing Status** | **Scientific Name** | **Common Name** | **Federal Listing Status** |
| --- | --- | --- | --- | --- | --- |
| **Plants (n = 105)** |  |  | **Invertebrates (n = 21)** |  |  |
| *Adiantum vivesii* | No common name | E | *Amblema neislerii* | Fat threeridge (mussel) | E |
| *Agave eggersiana* | No common name | E | *Anaea troglodyta floridalis* | Florida leafwing Butterfly | E |
| *Amaranthus pumilus* | Seabeach amaranth | T | *Cicindelidia floridana* | Miami tiger beetle | E |
| *Amorpha crenulata* | Crenulate lead-plant | E | *Cyclargus (=Hemiargus) thomasi bethunebakeri* | Miami Blue Butterfly | E |
| *Argythamnia blodgettii* | Blodgett's silverbush | T | *Cyclargus ammon* | Nickerbean Blue Butterfly | S |
| *Aristida chaseae* | No common name | E | *Elliptio chipolaensis* | Chipola slabshell | T |
| *Aristida portoricensis* | Pelos del diablo | E | *Elliptio spinosa* | Altamaha Spinymussel | E |
| *Asimina tetramera* | Four-petal pawpaw | E | *Elliptoideus sloatianus* | Purple bankclimber (mussel) | T |
| *Auerodendron pauciflorum* | No common name | E | *Epioblasma florentina walkeri (=E. walkeri)* | Tan riffleshell | E |
| *Banara vanderbiltii* | Palo de ramon | E | *Fusconaia burkei* | Tapered pigtoe | T |
| *Baptisia arachnifera* | Hairy rattleweed | E | *Fusconaia escambia* | Narrow pigtoe | T |
| *Bonamia grandiflora* | Florida bonamia | T | *Fusconaia rotulata* | Round Ebonyshell | E |
| *Brickellia mosieri* | Florida brickell-bush | E | *Hamiota australis* | Southern sandshell | T |
| *Buxus vahlii* | Vahl's boxwood | E | *Hemiargus ceraunus antibubastus* | Ceraunus Blue Butterfly | S |
| *Callicarpa ampla* | Capa rosa | E | *Heraclides aristodemus ponceanus* | Schaus swallowtail butterfly | E |
| *Calyptranthes thomasiana* | No common name | E | *Leptotes cassius theonus* | Cassius Blue Butterfly | S |
| *Calyptronoma rivalis* | Palma de manaca | T | *Medionidus walkeri* | Suwannee moccasinshell | T |
| *Campanula robinsiae* | Brooksville bellflower | E | *Orthalicus reses (not incl. nesodryas)* | Stock Island tree snail | T |
| *Carex lutea* | Golden sedge | E | *Pleurobema strodeanum* | Fuzzy pigtoe | T |
| *Catesbaea melanocarpa* | No common name | E | *Ptychobranchus jonesi* | Southern kidneyshell | E |
| *Cereus eriophorus*  var*. fragrans* | Fragrant prickly-apple | E | *Strymon acis bartrami* | Bartram's hairstreak Butterfly | E |
| *Chamaecrista glandulosa*  var*. mirabilis* | No common name | E |  |  |  |
| *Chamaecrista lineata keyensis* | Big Pine partridge pea | E |  |  |  |
| *Chamaesyce deltoidea pinetorum* | Pineland sandmat | T |  |  |  |
| *Chamaesyce deltoidea serpyllum* | Wedge spurge | E |  |  |  |
| *Chamaesyce deltoidea ssp. deltoidea* | Deltoid spurge | E |  |  |  |
| *Chamaesyce garberi* | Garber's spurge | T |  |  |  |
| *Chionanthus pygmaeus* | Pygmy fringe-tree | E |  |  |  |
| *Chromolaena frustrata* | Cape Sable Thoroughwort | E |  |  |  |
| *Chrysopsis floridana* | Florida golden aster | E |  |  |  |
| *Cladonia perforata* | Florida perforate cladonia | E |  |  |  |
| *Conradina glabra* | Apalachicola rosemary | E |  |  |  |
| *Consolea corallicola* | Florida semaphore Cactus | E |  |  |  |
| *Cordia bellonis* | No common name | E |  |  |  |
| *Cornutia obovata* | Palo de nigua | E |  |  |  |
| *Cranichis ricartii* | No common name | E |  |  |  |
| *Crescentia portoricensis* | Higuero de sierra | E |  |  |  |
| *Cucurbita okeechobeensis ssp. okeechobeensis* | Okeechobee gourd | E |  |  |  |
| *Cyathea dryopteroides* | Elfin tree fern | E |  |  |  |
| *Dalea carthagenensis floridana* | Florida prairie-clover | E |  |  |  |
| *Daphnopsis hellerana* | No common name | E |  |  |  |
| *Deeringothamnus pulchellus* | Beautiful pawpaw | E |  |  |  |
| *Deeringothamnus rugelii* | Rugel's pawpaw | E |  |  |  |
| *Dicerandra immaculata* | Lakela's mint | E |  |  |  |
| *Digitaria pauciflora* | Florida pineland crabgrass | T |  |  |  |
| *Elaphoglossum serpens* | No common name | E |  |  |  |
| *Eugenia haematocarpa* | Uvillo | E |  |  |  |
| *Eugenia woodburyana* | No common name | E |  |  |  |
| *Euphorbia telephioides* | Telephus spurge | T |  |  |  |
| *Galactia smallii* | Small's milkpea | E |  |  |  |
| *Gesneria pauciflora* | No common name | T |  |  |  |
| *Goetzea elegans* | Beautiful goetzea | E |  |  |  |
| *Gonocalyx concolor* | No common name | E |  |  |  |
| *Harperocallis flava* | Harper's beauty | E |  |  |  |
| *Harrisia (=Cereus) aboriginum (=gracilis)* | Aboriginal Prickly-apple | E |  |  |  |
| *Harrisia portoricensis* | Higo Chumbo | T |  |  |  |
| *Ilex cookii* | Cook's holly | E |  |  |  |
| *Ilex sintenisii* | No common name | E |  |  |  |
| *Isoetes louisianensis* | Louisiana quillwort | E |  |  |  |
| *Jacquemontia reclinata* | Beach jacquemontia | E |  |  |  |
| *Juglans jamaicensis* | West Indian Walnut (=Nogal) | E |  |  |  |
| *Justicia cooleyi* | Cooley's water-willow | E |  |  |  |
| *Lepanthes eltoroensis* | No common name | E |  |  |  |
| *Leptocereus grantianus* | No common name | E |  |  |  |
| *Lindera melissifolia* | Pondberry | E |  |  |  |
| *Linum arenicola* | Sand flax | E |  |  |  |
| *Linum carteri carteri* | Carter's small-flowered flax | E |  |  |  |
| *Lyonia truncata var. proctorii* | No common name | E |  |  |  |
| *Lysimachia asperulaefolia* | Rough-leaved loosestrife | E |  |  |  |
| *Macbridea alba* | White birds-in-a-nest | T |  |  |  |
| *Mitracarpus maxwelliae* | No common name | E |  |  |  |
| *Mitracarpus polycladus* | No common name | E |  |  |  |
| *Myrcia paganii* | No common name | E |  |  |  |
| *Nolina brittoniana* | Britton's beargrass | E |  |  |  |
| *Ottoschulzia rhodoxylon* | Palo de rosa | E |  |  |  |
| *Oxypolis canbyi* | Canby's dropwort | E |  |  |  |
| *Peperomia wheeleri* | Wheeler's peperomia | E |  |  |  |
| *Pilosocereus robinii* | Key tree cactus | E |  |  |  |
| *Pinguicula ionantha* | Godfrey's butterwort | T |  |  |  |
| *Pleodendron macranthum* | Chupacallos | E |  |  |  |
| *Polygala lewtonii* | Lewton's polygala | E |  |  |  |
| *Polygala smallii* | Tiny polygala | E |  |  |  |
| *Polystichum calderonense* | No common name | E |  |  |  |
| *Rhododendron chapmanii* | Chapman rhododendron | E |  |  |  |
| *Schoepfia arenaria* | No common name | T |  |  |  |
| *Schwalbea americana* | American chaffseed | E |  |  |  |
| *Scutellaria floridana* | Florida skullcap | T |  |  |  |
| *Sideroxylon reclinatum ssp. austrofloridense* | Everglades bully | T |  |  |  |
| *Solanum drymophilum* | Erubia | E |  |  |  |
| *Stahlia monosperma* | Cobana negra | T |  |  |  |
| *Styrax portoricensis* | Palo de jazmin | E |  |  |  |
| *Tectaria estremerana* | No common name | E |  |  |  |
| *Ternstroemia luquillensis* | Palo colorado | E |  |  |  |
| *Ternstroemia subsessilis* | No common name | E |  |  |  |
| *Thalictrum cooleyi* | Cooley's meadowrue | E |  |  |  |
| *Thelypteris inabonensis* | No common name | E |  |  |  |
| *Thelypteris verecunda* | No common name | E |  |  |  |
| *Thelypteris yaucoensis* | No common name | E |  |  |  |
| *Trichilia triacantha* | Bariaco | E |  |  |  |
| *Trichomanes punctatum ssp. floridanum* | Florida bristle fern | E |  |  |  |
| *Varronia rupicola* | No common name | T |  |  |  |
| *Vernonia proctorii* | No common name | E |  |  |  |
| *Villosa choctawensis* | Choctaw bean | E |  |  |  |
| *Warea carteri* | Carter's mustard | E |  |  |  |
| *Zanthoxylum thomasianum* | St. Thomas prickly-ash | E |  |  |  |

Table S3. Federally-listed vertebrate species that occur in hurricane-prone regions of the Caribbean and the southeastern United States. Species are categorized as to the status of their recovery plan. If a plan exists, whether it mentions hurricanes as a threat, as well as whether it discusses management actions in response to hurricanes, is also indicated. E = Endangered; T = Threatened; S = Similarity of appearance to a threatened taxon; DPS = Distinct Population Segment; RP = Recovery Plan.

| **Scientific Name** | **Common Name** | **Federal Listing Status** | **Final Recovery Plan?**  **(Date)** | **Threat of Hurricane Mentioned in Plan?** | **Management Actions Discussed in Plan?** |
| --- | --- | --- | --- | --- | --- |
| **Fishes (n = 2)** |  |  |  |  |  |
| *Acipenser oxyrinchus (=oxyrhynchus) desotoi* | Atlantic sturgeon  (Gulf subspecies) | T | Yes (1995) | No | No |
| *Etheostoma okaloosae* | Okaloosa darter | T | Yes (1998) | Yes | No. RP states that “the darter population…persisted through… hurricanes in 1995” |
|  |  |  |  |  |  |
| **Amphibians (n = 6)** |  |  |  |  |  |
| *Ambystoma bishopi* | Reticulated Flatwoods Salamander | E | Draft  (2018) | Yes | Yes. See below for *A. cingulatum* |
| *Ambystoma cingulatum* | Frosted Flatwoods salamander | T | Draft (2018) | Yes | Yes. Recovery Implementation Strategy states that “the St. Marks population is especially susceptible to saltwater intrusion from hurricanes or sea level rise.” Land acquisition, translocations and ex situ conservation (captive breeding program) are mentioned as recovery actions, although not specifically in reference to hurricanes. |
| *Eleutherodactylus cooki* | Guajon | T | Yes (2004) | Yes | Yes |
| *Eleutherodactylus jasperi* | Golden coqui | T | Yes (1984) | No | No |
| *Eleutherodactylus juanariveroi* | Llanero Coqui | E | No |  |  |
| *Peltophryne lemur* | Puerto Rican crested toad | T | Yes (1992) | Yes | No |
|  |  |  |  |  |  |
| **Reptiles (n = 21)** |  |  |  |  |  |
| *Alligator mississippiensis* | American alligator | S | N/A |  |  |
| *Ameiva polops* | St. Croix ground lizard | E | Yes (1984) | No | No |
| *Anolis roosevelti* | Culebra Island giant anole | E | Yes (1983) | No | No |
| *Caretta caretta*  (Northwest Atlantic Ocean DPS) | Loggerhead sea turtle | T | Yes (2009) | Yes | Limited, and not discussed with reference to hurricanes. Suitable actions taken include land nesting habitat acquisition. RP states that “sea turtles have evolved a strategy to offset these natural events [tropical storms and hurricanes] by laying large numbers of eggs and by distributing their nests both spatially and temporally. Thus, the total annual hatchling production is never fully affected by storm-generated beach erosion and inundation, although local effects may be high.” More emphasis on modeling effects of sea level rise on nesting beaches. |
| *Chelonia mydas*  (North Atlantic Ocean DPS) | Green sea turtle | T | Yes (1991) | Yes | No. Yet, severe storm events (e.g., tropical storms, hurricanes) may result in significant nest loss although, in the SE US, such storms generally occur after the peak of the hatching season. Inundation of nests and accretion of sand above incubating nests as a result of a late season storm played a major role in destroying nests from which hatchlings had not yet emerged. |
| *Chelonia mydas*  (South Atlantic Ocean DPS) | Green sea turtle | T | Yes (1991) | Yes | No. See above for North Atlantic Ocean DPS |
| *Crocodylus acutus* | American crocodile | T | Yes (1999) | Yes | No. Yet, hurricanes are thought to adversely affect American crocodiles and may be one of the most important factors limiting the number and distribution of this species in South Florida. Crocodiles undoubtedly perish during tropical storms and hurricanes that make landfall in extreme South Florida. The tidal surges, rough seas, and high winds probably result in direct mortality, but may also erode important nesting beaches, destroy nests, and alter other important habitat features. |
| *Cyclura stejnegeri* | Mona ground Iguana | T | Yes (1984) | No | No |
| *Dermochelys coriacea* | Leatherback sea turtle | E | Yes (1992) | Yes | No. Yet, leatherbacks nest in the tropics during hurricane season when the potential exists for storm generated waves and wind to erode nesting beaches, resulting in nest loss. Twelve nests were lost to Hurricane Dean in 1989 on Culebra. In 1980, only four out of approximately 80 nests laid on Sandy Point NWR survived to hatch following the catastrophic effects of Hurricane Allen. |
| *Drymarchon corais couperi* | Eastern indigo snake | T | Yes (1982) | No | No |
| *Epicrates inornatus* | Puerto Rican boa | E | Yes (1986) | No | No |
| *Epicrates monensis granti* | Virgin Islands tree boa | E | Yes (1983) | Yes | No |
| *Epicrates monensis monensis* | Mona boa | T | Yes  (1984) | No | No |
| *Eretmochelys imbricata* | Hawksbill sea turtle | E | Yes (1993) | No | No |
| *Gopherus polyphemus*  (western population) | Gopher tortoise | T | Yes  (1990) | No | No |
| *Graptemys flavimaculata* | Yellow-blotched map turtle | T | Yes (1993) | No | No |
| *Graptemys oculifera* | Ringed map turtle | T | Yes (1988) | No | No |
| *Nerodia clarkii taeniata* | Atlantic salt marsh snake | T | Yes (1993) | No | No |
| *Pituophis melanoleucus lodingi* | Black pine snake | T | No |  |  |
| *Pseudemys alabamensis* | Alabama red-bellied turtle | E | Yes (1990) | No | No |
| *Sphaerodactylus micropithecus* | Monito gecko | E | Yes (1986) | No | No |
|  |  |  |  |  |  |
| **Birds (n = 20)** |  |  |  |  |  |
| *Accipiter striatus venator* | Puerto Rican sharp-shinned hawk | E | Yes (1997) | Yes | No. Declines attributed to Hurricane Hugo in 1989 are disputed |
| *Agelaius xanthomus* | Yellow-shouldered blackbird | E | Yes (1996) | Yes | No. Yet, Hurricane Hugo (1989) is believed to have extirpated a population of critically low abundance |
| *Amazona vittata* | Puerto Rican parrot | E | Yes (2009) | Yes | No. Yet, Hurricane Hugo (1989) reduced wild population size from 47 to 23 individuals; second population established, otherwise, none |
| *Ammodramus maritimus mirabilis* | Cape Sable seaside sparrow | E | Yes (1999) | Yes | No. Yet, the Great Labor Day Hurricane of 1935 is thought to have initiated vegetative changes in the Cape Sable area that eventually led to extirpation of the Cape Sable population of the sparrow |
| *Ammodramus savannarum floridanus* | Florida grasshopper sparrow | E | Yes (1999) | No | No |
| *Aphelocoma coerulescens* | Florida scrub-jay | T | Yes (1999) | No | No |
| *Buteo platypterus brunnescens* | Puerto Rican broad-winged hawk | E | Yes (1997) | Yes | No |
| *Campephilus principalis* | Ivory-billed woodpecker | E | Yes (2010) | No | No |
| *Caprimulgus noctitherus* | Puerto Rican nightjar | E | Yes (1984) | No | No |
| *Columba inornata wetmorei* | Puerto Rican plain Pigeon | E | Yes (1982) | Yes | No |
| *Corvus leucognaphalus* | White-necked crow | E | No |  |  |
| *Grus canadensis pulla* | Mississippi sandhill crane | E | Yes (1991) | Yes | No |
| *Mycteria americana* | Wood stork | T | Yes (1999) | No | No |
| *Picoides borealis* | Red-cockaded woodpecker | E | Yes (2003) | Yes | Yes. Recovery plan states that “Hurricanes are the greatest catastrophic threat to population viability,” and identified Hurricane Hugo (1989) as cause of “catastrophic changes in mortality” in the Francis Marion National Forest, SC. |
| *Polyborus plancus audubonii* | Audubon's crested caracara | T | Yes (1999) | No | No |
| *Pterodroma cahow* | Bermuda petrel | E | No |  |  |
| *Rostrhamus sociabilis plumbeus* | Everglade snail kite | E | Yes (1999) | No | No |
| *Setophaga angelae* | Elfin-woods warbler | T | No |  |  |
| *Sterna dougallii dougallii* | Roseate tern | T | Yes (1999) | Yes | No |
| *Vermivora bachmanii* | Bachman's warbler (=wood) | E | Exempt from recovery planning |  |  |
|  |  |  |  |  |  |
| **Mammals (n = 16)** |  |  |  |  |  |
| *Canis rufus* | Red wolf | E | Yes (1990) | Yes | No. Yet, Hurricane Hugo played a role in death of one of the original wolves released in 1989 |
| *Eumops floridanus* | Florida bonneted bat | E | No |  |  |
| *Microtus pennsylvanicus dukecampbelli* | Florida salt marsh vole | E | Yes (1997) | Yes | No. Yet, effects of Hurricane Andrew and Hurricane Georges – in combination with other factors – may have reduced the population below a minimum threshold needed to rebound. |
| *Neotoma floridana smalli* | Key Largo woodrat | E | Yes (1999) | Yes | Limited. Public land acquisition |
| *Odocoileus virginianus clavium* | Key deer | E | Yes (1999) | Yes | Limited. filling of ditches to limit influx of saline water |
| *Oryzomys palustris natator* | Rice rat | E | Yes  (1999) | Yes | No |
| *Peromyscus gossypinus allapaticola* | Key Largo cotton mouse | E | Yes (1999) | Yes | No |
| *Peromyscus polionotus allophrys* | Choctawhatchee beach mouse | E | Yes (1987) | Yes | No |
| *Peromyscus polionotus ammobates* | Alabama beach mouse | E | Yes (1987) | Yes | No |
| *Peromyscus polionotus niveiventris* | Southeastern beach mouse | T | Yes (1999) | Yes | No |
| *Peromyscus polionotus peninsularis* | St. Andrew beach mouse | E | Yes (2010) | Yes | Yes. Emergency response plan (for hurricanes) is required for downlisting; successful translocation and re-establishment of a second population |
| *Peromyscus polionotus phasma* | Anastasia Island beach mouse | E | Yes (1993) | Yes | No |
| *Peromyscus polionotus trissyllepsis* | Perdido Key beach mouse | E | Yes (1987) | Yes | No |
| *Puma (=Felis) concolor coryi* | Florida panther | E | Yes (2008) | No | No |
| *Sylvilagus palustris hefneri* | Lower Keys marsh rabbit | E | Yes (1999) | No | No |
| *Trichechus manatus* | West Indian Manatee | T | Yes (2001) | Yes | No |
